# Increasing productivity of hybridoma cell lines by sorting by side scattering light

**DOI:** 10.1186/1753-6561-5-S8-P83

**Published:** 2011-11-22

**Authors:** Daniel Landgrebe, Cornelia Kasper, Thomas Scheper

**Affiliations:** 1 Institute for Technical Chemistry, Leibniz University of Hannover, 30167 Hannover, Germany

## Introduction

The side scattering light of a mammalian cell is caused, among other things, by the membranes of cell organelles. We suggest that cells with a high side scatter contain a large amount of mitochondria and a large endoplasmatic reticulum. In a simple approach we separate cells with a high side scatter via fluorescence activated cell sorting (FACS) to create sub populations with a higher productivity. We obtain cells with a high amount of mitochondria and a large endoplasmatic reticulum which could cause strong energy metabolism and high protein productivity. The advantage of this technique is that no staining dye or complex procedure is needed to reach the goal of increasing the productivity of a cell line.

## Materials and methods

For the sorting procedure the SC-71 murine hybridoma cell line (DSMZ, Braunschweig) is used. The cells produce an IgG_1_ specific for rat myosin. Cultivation of the cells before and after the sorting steps was performed at the same conditions. The cells were grown in 100 ml Erlenmeyer shacking flasks with 20 ml medium (DMEM (Sigma-Aldrich, Steinheim),10 % FCS, 4mM glutamine, 1 % Penicillin/Streptomycin solution (PAA, Cölbe) ; conditions: 37°, 5 % CO_2_, 110 rpm) with an inoculation density of 0.45*10^6^ cells/ml. During the cultivation samples were taken twice a day for the acquisition of the cell number and offline analysis (product concentration via ELISA (Roche, Mannheim), glucose and lactate via YSI 2700 Biochemistry Analyzer (Yellow Springs Instruments) and amino acids via HPLC (Agilent Technologies, Waldbronn)). The product concentration is used to calculate the specific productivity rate of the cell lines as described by Brezinsky *et al*. [1].

For each sorting 5*10^6^ cells were taken from the exponential phase of a preparatory culture. After a washing step with PBS the cells were stained with Propidium iodide (5 µl [1 mg/ml] (Sigma-Aldrich, Steinheim)). Only Propidium iodide negatives cells were sorted to exclude dead cells. Aggregates were excluded by analyzing the pulse heights and their pulse integrals. For the separation cells with the maximal 3 % of the side scatter were sorted into a 6 well plate with 1ml of DMEM using a FACSVantage (BD Biosciences, Heidelberg) equipped with pulse processing. After an expansion time of 4 weeks the cells were used to repeat the procedure and to investigate growth and productivity qualities. The procedure were repeated three times to generate altogether four cell lines, one initial cell line and three sub cell lines (named population I – III according to the repeated sorting procedures).

Subsequent flow cytometric analyses were used to verify the constancy of the sorting effect. Therefore the four populations were cultivated for two month. Passages were performed by medium changes every third or fourth day. Samples were taken after the twenties passage. 0.5*10^6^ cells of each cell line were washed with PBS and the side scatter was analyzed using an Epics XL-MCL flow cytometer (Beckman Coulter, Krefeld).

## Results

**Figure 1:**
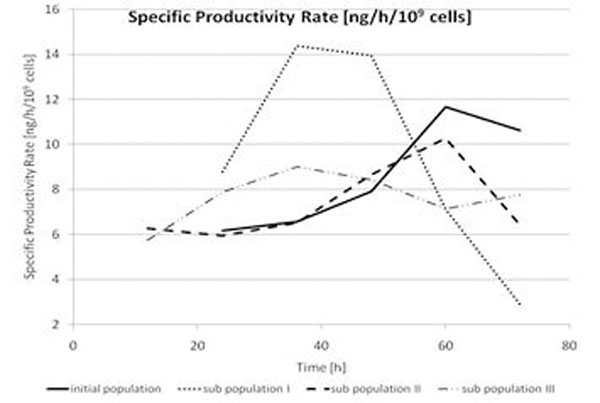
Specific productivity rates of initial population and sub populations

The calculation of the specific productivity rates show that a higher specific productivity is achieved earlier in the sub populations than in the initial population (Fig. 1). Nevertheless the total productivity is the same. Sub populations I and III show a maximal specific productivity already after 36 h (resp. after 60 h for sub population II). Compared with the initial population which reaches their max. after 72 h, it is a considerable increase of a important process parameter. The sub populations show also an increased cell density of 10 to 30 %. This effect is retrograding in the later sub populations. The cytometric analyses of the long term cultivation show that the separated cells have kept an increased side scatter. This effect is stable for at least 2 month of cultivation.

## Conclusions

The results suggest that the sub populations have a modified metabolism. This could be a result of the accumulation of mitochondria. To confirm this further experiments are necessary. These experiments should include staining of mitochondria and endoplasmatic reticulum and subsequent flow cytometric analysis to determine the amount of these organelles. Further investigations for the consumption of the main metabolites glucose and glutamate and the production of lactate could explain the changes in the growth behavior and productivity of the cells.
